# Gummi und andere Materialien: BfR-Empfehlungen zu Materialien im Lebensmittelkontakt

**DOI:** 10.1007/s00103-024-03938-x

**Published:** 2024-08-27

**Authors:** Suzan Fiack, Sarah von Leliwa, Thomas Tietz, Friederike Kühne

**Affiliations:** 1 Abteilung Risikokommunikation, Bundesinstitut für Risikobewertung, Max-Dohrn-Str. 8–10, 10589 Berlin, Deutschland; 2 Abteilung Chemikalien- und Produktsicherheit, Bundesinstitut für Risikobewertung, Max-Dohrn-Str. 8–10, 10589 Berlin, Deutschland

Seit 1958 veröffentlicht das Bundesinstitut für Risikobewertung (BfR), bzw. dessen Vorläuferinstitutionen, Empfehlungen zur gesundheitlichen Bewertung von Materialien für den Lebensmittelkontakt. Es handelt sich hierbei um Positivlisten zu Materialien, die nicht durch europäische oder nationale Gesetzgebung geregelt sind. Dazu zählen unter anderem Lebensmittelkontaktmaterialien aus Gummi, Silikon oder Papier sowie Produkte wie Kunstdärme oder Beschichtungen für Pfannen und Waffeleisen.

Die BfR-Empfehlungen sind keine Rechtsnormen. Sie stellen aber den derzeitigen Stand von Wissenschaft und Technik für die Bedingungen dar, unter denen Lebensmittelbedarfsgegenstände entsprechend der gesetzlichen Regelungen keine Stoffe in Mengen abgeben, die geeignet sind, die menschliche Gesundheit zu gefährden. Zudem sind auch einige Vorgaben enthalten, die sicherstellen sollen, dass in Kontakt kommende Lebensmittel nicht in ihrer Zusammensetzung unvertretbar verändert oder in ihren organoleptischen, d. h. mit den Sinnen wahrnehmbaren Eigenschaften beeinträchtigt werden.

Um einer europäischen Harmonisierung Vorschub zu leisten, richtet sich die Risikobewertung des BfR für neu aufzunehmende Substanzen nach Kriterien, die von der Europäischen Behörde für Lebensmittelsicherheit (EFSA) für die Bewertung eines Stoffes, der in Lebensmittelkontaktmaterialien aus Kunststoff eingesetzt werden soll, vorgeschrieben werden. Dies betrifft die Charakterisierung der Substanz, Informationen zu ihrer Herstellung und Verwendung, Daten zum Übergang der Substanz auf Lebensmittel und zu ihren toxikologischen Eigenschaften. Die Risikobewertung umfasst dabei nicht nur die Substanz selbst, sondern auch Verunreinigungen sowie Abbau- und Reaktionsprodukte – sogenannte „non-intentionally added substances“ (NIAS).

Die BfR-Empfehlungen zu Materialien für den Lebensmittelkontakt sind dynamische Dokumente, die ständig weiterentwickelt und aktualisiert werden, um dem aktuellen Stand der Technik und der Risikobewertung zu entsprechen. So werden bestehende Substanzen gestrichen oder neue gelistet, Prüfbedingungen aktualisiert oder Verwendungsbedingungen von Substanzen an aktuelle Bewertungsmaßstäbe und neue Daten angepasst.

In der vorliegenden Ausgabe des *Bundesgesundheitsblatts* werden die überarbeiteten Empfehlungen XXI, XXI/1 und XXI/2 zu Bedarfsgegenständen auf Basis von Natur- und Synthesekautschuk – weithin als „Gummiempfehlungen“ bezeichnet – veröffentlicht. Gummi tritt im Alltag als Lebensmittelkontaktmaterial zwar nicht so prominent in Erscheinung wie Kunststoff oder Papier und findet eher in weniger auffälligen Anwendungen wie Dichtringen, Handschuhen und Beruhigungssaugern Verwendung. Dennoch sollte seine Bedeutung nicht unterschätzt werden, da Gummi in der Lebensmittelherstellung durchaus großflächig mit Lebensmitteln in Kontakt kommt, etwa in Melkanlagen, Förderbändern und Schläuchen.

Bereits im Juli 2021 wurde eine weitreichende Überarbeitung der Empfehlung XXI durchgeführt, die eine Neueinführung der Unterempfehlungen XXI/1 und XXI/2 beinhaltete. Die Empfehlung XXI ist als definierende Rahmenempfehlung zu betrachten und enthält alle vollständig bewerteten Substanzen. Die Empfehlung XXI/1 bezieht sich auf Bedarfsgegenstände aus Gummi im Kontakt mit Lebensmitteln. Je nach Kontaktzeit werden 4 verschiedene Kategorien und entsprechende Prüfbedingungen unterschieden. Die Empfehlung XXI/2 bezieht sich auf Bedarfsgegenstände, die in Kontakt mit der Mundschleimhaut kommen, wie z. B. Ernährungs- und Beruhigungssauger.

Sowohl Empfehlung XXI/1 als auch XXI/2 enthalten Listen mit Substanzen, die seit Langem zur Herstellung von Gummi verwendet werden, deren gesundheitliche Bewertung aber nicht dem aktuellen Stand entspricht. Im Bestreben, die Substanzen zu bewerten bzw. nicht benötigte Substanzen von der Liste zu streichen, waren Hersteller aufgefordert, Stoffe zu benennen, für die sie vollständige neue Antragsdaten einreichen werden. Substanzen, für die eine solche Absichtserklärung nicht eingereicht wurde, sind mit der vorliegenden Veröffentlichung gestrichen worden. Dies führte z. B. zu einer erheblichen Reduktion der Anzahl der empfohlenen Vernetzungsmittel. Zudem wurden einige vormals sehr allgemeine bzw. generische Einträge deutlich konkretisiert. Weitere Neuerungen beziehen sich auf die Prüfbedingungen für Kappen, Dichtungen und Verschlüsse. Die vorliegende Neuveröffentlichung stellt damit einen wichtigen Schritt in Richtung einer modernen Risikobewertung für Bedarfsgegenstände aus Gummi dar.

Im Sinne des gesundheitlichen Verbraucherschutzes wünschen wir Ihnen viel Freude bei der Lektüre dieser Ausgabe des *Bundesgesundheitsblatts*.

